# Nutritional Challenges among African Refugee and Internally Displaced Children: A Comprehensive Scoping Review

**DOI:** 10.3390/children11030318

**Published:** 2024-03-07

**Authors:** Claire Gooding, Salwa Musa, Tina Lavin, Lindiwe Sibeko, Chizoma Millicent Ndikom, Stella Iwuagwu, Mary Ani-Amponsah, Aloysius Nwabugo Maduforo, Bukola Salami

**Affiliations:** 1 Centre for Health Services Research, School of Population and Global Health, University of Western Australia, 35 Stirling Hwy, Crawley, WA 6009, Australia; claire.gooding@uwa.edu.au (C.G.); tina.lavin@uwa.edu.au (T.L.); 2 Faculty of Nursing, University of Alberta, Edmonton, AB T6G 1C9, Canada; smusa1@ualberta.ca; 3 Western Australia Centre for Rural Health, School of Population and Global Health, University of Western Australia, 35 Stirling Hwy, Crawley, WA 6009, Australia; 4 Department of Nutrition, School of Public Health and Health Sciences, University of Massachusetts Amherst, Amherst, MA 01003, USA; lsibeko@umass.edu; 5 Department of Nursing, College of Medicine, University of Ibadan, Ibadan 200005, Nigeria; cmndikom@com.ui.edu.ng; 6 Centre for the Right to Health, Abuja 904101, Nigeria; siwuagwu@crhnigeria.org; 7 Maternal and Child Health Department, School of Nursing and Midwifery, University of Ghana, Accra P.O. Box LG43, Ghana; mnkansah@ualberta.ca; 8 Department of Community Health Science, Cumming School of Medicine, University of Calgary, Calgary, AB T2N 1N4, Canada; oluwabukola.salami@ucalgary.ca

**Keywords:** malnutrition, vulnerable population, food security, children

## Abstract

Background: Children’s nutrition in Africa, especially among those displaced by conflicts, is a critical global health concern. Adequate nutrition is pivotal for children’s well-being and development, yet those affected by displacement confront distinctive challenges. This scoping review seeks to enhance our current knowledge, filling gaps in understanding nutritional and associated health risks within this vulnerable population. Objective: We conducted a scoping review of the literature on the nutritional status and associated health outcomes of this vulnerable population with the goal of informing targeted interventions, policy development, and future research efforts to enhance the well-being of African refugee and internally displaced children. Methods: This scoping review adopted Arksey and O’Malley (2005)’s methodology and considered studies published between 2000 and 2021. Results: Twenty-three published articles met the inclusion criteria. These articles highlighted a wide variation in the levels of malnutrition among African refugee/internally displaced (IDP) children, with the prevalence of chronic malnutrition (stunting) and acute malnutrition (wasting) ranging from 18.8 to 52.1% and 0.04 to 29.3%, respectively. Chronic malnutrition was of ‘high’ or ‘very high’ severity (according to recent WHO classifications) in 80% of studies, while acute malnutrition was of ‘high’ or ‘very high’ severity in 50% of studies. In addition, anemia prevalence was higher than the 40% level considered to indicate a severe public health problem in 80% of the studies reviewed. Conclusion: In many settings, acute, chronic, and micronutrient malnutrition are at levels of great concern. Many countries hosting large, displaced populations are not represented in the literature, and research among older children is also lacking. Qualitative and intervention-focused research are urgently needed.

## 1. Introduction

The 2023 statistics and trends report giving a regional overview of food security and nutrition in Africa [1] reported that Africa is facing an unprecedented food crisis, with nearly 20% of the population, around 282 million people, experiencing undernourishment—an increase of 57 million since the COVID-19 pandemic. Over one billion Africans cannot afford a healthy diet, and approximately 30% of children suffer from stunting due to malnutrition. The continent is falling behind on its food security targets for 2030, and malnutrition remains a significant challenge. Despite some positive aspects, such as high rates of exclusive breastfeeding, the majority (78%) cannot afford a healthy diet. The rising cost of a healthy diet is rendering it unaffordable for both the poor and a substantial non-poor population.

On the other hand, the United Nations High Commissioner for Refugees (UNHCR) reported that the number of refugees and internally displaced persons (IDPs) reached an unprecedented 82.4 million worldwide in 2020, of whom 31.8 million (38%) were in Africa [2]. In Sub-Saharan Africa, children under 18 years of age represent 57% of this population of concern, with levels as high as 62–63% in Somalia, South Sudan, the Central African Republic, and the Democratic Republic of Congo (DRC) [2]. Children affected by displacement are vulnerable to undernutrition due to food insecurity, often exacerbated by poor environmental, sanitation, and shelter conditions [3]. Nearly 50% of deaths in children under the age of five are attributed to malnutrition [4], and those who survive face short- and long-term negative impacts related to their physiological, cognitive, emotional, and social development [5].

Malnutrition is commonly characterized as either acute, encompassing conditions such as wasting (where weight for height falls more than 2 standard deviations below the World Health Organization Child Growth Standards median), or chronic, which involves stunting (height for age more than 2 standard deviations below the WHO Child Growth Standards median) [6]. ‘Underweight’ (weight for age > 2 SD below the WHO CGS median) is a composite indicator that can be a result of wasting, stunting, or both [6]. A meta-analysis of surveys examining the demographics and health of children under the age of 5 in 32 Sub-Saharan African countries revealed an overall prevalence of stunting at 33.2%, ranging from 16.5% in Gabon to 57.7% in Burundi. Additionally, the overall prevalence of wasting was 7.1%, varying from 2.2% in Rwanda to 15.5% in Burkina Faso, while the overall prevalence of underweight was 16.3%, ranging from 1.5% in Zambia to 36.4% in Niger [7]. A systematic review was also conducted to determine the factors associated with child malnutrition in Sub-Saharan Africa [8]. The authors found low parent education levels, low socioeconomic status, rural residence, poor source of drinking water, maternal underweight, young maternal age, low birth weight, breastfeeding > 12 months, diarrheal episodes, male sex, and increasing age were associated with stunting, wasting, and underweight in children under 5 years old. The overall prevalence of undernourishment in Africa has been on a steady rise since 2015, particularly witnessing sharp increases in countries affected by conflict [9].

Studies indicate that in 2016, 2.6 million individuals within Africa experienced internal displacement, while approximately 5.4 million people were recognized as refugees from the continent [10,11]. The cause of displacement in Africa has been attributed to political violence, banditry, terrorism, environmental calamities like floods or droughts, and compounded elements such as the escalating famine and drought emergency [1,11,12]. Despite growing numbers of children in Africa affected by displacement and evidence of concerning levels of malnutrition in the region, to our knowledge, no attempt has yet been made to synthesize the relevant literature on this particularly vulnerable population. The objective of this scooping review is to identify, summarize, and analyze the key findings related to the nutritional status and health outcomes of this vulnerable population. By synthesizing the available evidence, this research intends to provide a nuanced understanding of the multifaceted factors influencing the nutritional well-being of African refugees and internally displaced children. Ultimately, the insights gained from this study can inform targeted interventions, policy development, and future research endeavors aimed at improving the nutritional outcomes and overall health of these marginalized children in the context of forced displacement.

## 2. Methods

This scoping review aimed to identify all relevant studies regardless of study design and utilized an iterative non-linear reflective process to ensure that the literature was comprehensively covered. Arksey and O’Malley [13] outline a five-stage process for scoping reviews: Stage 1, develop research question; Stage 2, identify relevant studies; Stage 3, article selection; Stage 4, data charting and data extraction; and Stage 5, summarize and report the results.

In Stage 1, we adopted the perspective of Levac, Colquhoun, and O’Brien (Levac et al., 2010) [14] to investigate a broad research question with a clear scope of inquiry. Our three main research questions were as follows:

(1) What is the scope, range, and nature of the evidence on the nutritional status/needs of African refugee and internally displaced children?
(2) What insights can be drawn from the current global literature concerning the nutritional status and needs of African refugee and internally displaced children?
(3) What future studies are needed to address any gaps in the knowledge identified?

**Figure 1:**
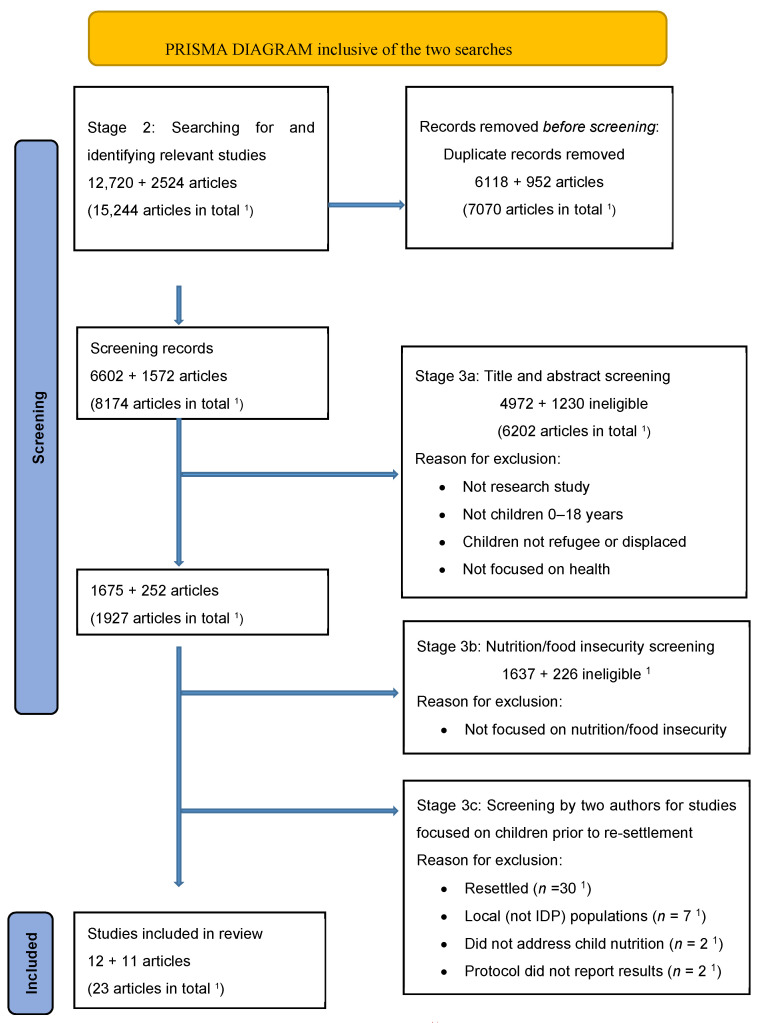
Flow chart of article screening. ^1^ the PRISMA diagram is inclusive of the two searches at each stage, Search 1 and Search 2.

Stage 2 involved searching for and identifying relevant studies in electronic databases, reference lists of relevant reviewed articles, and libraries of relevant organizations. A health services librarian conducted searches on the following databases on 21 January 2019 and 13 October 2021: Ovid MEDLINE^®^ and Epub Ahead of Print, In-Process & Other Non-Indexed Citations and Daily (1946 to current); Embase (1974 to current); Ovid Global Health (1910–Current); Ovid PsycInfo (1806–current); Cochrane Library (1993–present); EBSCO CINAHL Plus with Full-text (1937–current); EBSCO SocIndex (1895–present); EBSCO Child Development & Adolescent Studies (1927–present); ProQuest Sociological Abstracts (1952–present); and ProQuest Dissertations & Theses Global (1997–present). This scoping review was part of a larger study investigating the health of African children in the context of migration and displacement including after re-settlement. Therefore, the initial article search involved broad search terms: child health services OR adolescent health services OR child care OR infant care OR infant, newborn, diseases OR infant health OR child welfare OR infant welfare OR child nutrition disorders OR infant nutrition disorders OR child nutritional physiological phenomena OR adolescent nutritional physiological phenomena OR adolescent development OR adolescent health OR adolescent medicine OR adolescent psychiatry OR psychology, adolescent OR child psychiatry OR adolescent development OR child development OR psychology, child OR (child or children or childhood or infant* or newborn* or neonat* or baby or babies or preschool* or toddler* or adolescen* or teen* or youth* or pediatric* or paediatric*). These search terms were combined with emigrants and immigrants OR refugees OR (immigra* or refugee* or asylum seeker* or migrant* or displaced or displacement or transients) or (immigrant* or refugee*). These two groups of search terms were used in conjunction with Africa and the names of all African countries, with the search focusing on studies published between January 2000 and January 2019 (first search) and January 2019 and October 2021 (second search). The two searches returned 12,720 and 2524 records, respectively, which were exported into Covidence. After duplicates were removed, 6602 and 1572 records remained from the first and second searches, respectively. Figure 1 illustrates the screening process for articles.

Stage 3a involved title and abstract screening to select articles that met the following criteria: research studies; focused on children aged 0 to 18 years; child must be refugee or displaced person; and focused on health. Systematic reviews, literature reviews, conference abstracts and proceedings, articles that focused on the mother’s health only, and articles that focused on the experiences of parents (and not information on the health of the child) were excluded. This screening returned 1675 and 252 articles from the two respective searches. These records were then screened for nutrition studies that included the following terms in the title, abstract, or keywords: nutrition or food insecurity or food security or malnutrition or micronutrient or macronutrient or stunting or wasting or growth (Stage 3b). This additional screening returned 38 articles from the first search and 26 articles from the second search. Three authors (TL, CG, and SM) reviewed each full text independently with respect to the inclusion criteria for the scoping review, ensuring that the study population was children prior to re-settlement (i.e., refugee camp, informal settlement, other setting) (Stage 3c). Consensus was reached between authors where discrepancies occurred. Forty-one articles were excluded during the full article assessment as they focused on resettled children (*n* = 30), local (not IDP) populations (*n* = 7), did not address child nutrition (*n* = 2), or did not present results (i.e., study protocol; *n* = 2).

Stage 4 involved extracting the following information from each article: author name, title, year of publication, research questions or objectives, methodology, theoretical framework, method (sampling, sample size, age of child, data source—parent vs. child vs. health professional), clinical area of focus (e.g., mental health, nutrition, cardiovascular health), period of data collection, country of origin or region, destination country or region, summary of findings, and summary of implications.

Stage 5 involved the authors working collaboratively to identify recurring patterns and topics addressed across the selected studies. Through an iterative process, they organized these patterns into overarching themes that encapsulated the diverse aspects of child health in the context of migration and displacement.

Stage 6 involved summarizing, in the form of tables and text, and reporting the results through an iterative process involving the authors collaboratively discussing and refining the results.

## 3. Results

A combined total of 23 published articles from both searches met the inclusion criteria for analysis in this scoping review. The included studies were published between 2000 and 2021 in national and internationally recognized journals focusing on public health, pediatric health, and nutrition. Twenty-one studies employed a quantitative methodology, while one study utilized a qualitative approach, and another study employed mixed methods. Most (*n* = 18) studies were cross-sectional in nature, one used an observational design, and four evaluated an intervention. The predominant clinical area of focus was malnutrition (stunting, underweight, and wasting; *n* = 14), followed by micronutrient levels (*n* = 8) and newborn health (*n* = 1). The studies primarily used physiological data (*n* = 20), questionnaires (*n* = 12), interviews (*n* = 6), and focus groups (*n* = 2) to address their research objectives. The included studies are outlined in Table 1.

Four studies used non-probabilistic sampling methods (purposive/convenience), seven studies used two- or multi-stage randomized cluster sampling, three studies used systematic random sampling, two studies reported using cluster sampling (probability proportional to size), four studies used simple random sampling, and three quantitative studies did not report the sampling method. The sample sizes ranged from 18 to 5121 and most often included children aged 6–59 months (*n* = 14), followed by 3–59 months (*n* = 1), 6–23 months (*n* = 1), 3 months–10 years (*n* = 1), 4–13 years (*n* = 1), 10–13 years (*n* = 1), and 10–19 years (*n* = 2); other informants included parents, caregivers, family members, health program managers, front-line health workers, and public health officials (*n* = 2). The research was conducted in countries including Ethiopia (*n* = 4), Democratic Republic of the Congo (*n* = 2), Cameroon (*n* = 1), Chad (*n* = 1), Uganda (*n* = 2), Somalia (*n* = 4), Kenya (*n* = 1), Nigeria (*n* = 3), and South Sudan (*n* = 2). One study was conducted across five countries: Kenya, Uganda, Ethiopia, Algeria, and Zambia. One study analyzed retrospective data from 24 countries. One study investigated African asylum seekers before their resettlement in Israel.

The articles considered in this review are discussed below according to the three themes they address: malnutrition—stunting, wasting, and underweight; malnutrition—micronutrient imbalances; and neonatal health.

### 3.1. Malnutrition: Stunting, Wasting, and Underweight

Fifteen articles specifically focused on malnutrition, with fourteen using quantitative methods and one using mixed methods (see Table 1). The countries of interest included Chad, Somalia, Ethiopia, Uganda, Nigeria, Cameroon, and the Democratic Republic of the Congo. Of the 15 quantitative studies, 12 used cross-sectional surveys incorporating anthropometric measurements including weight, height, presence of edema, and middle-upper arm circumference (MUAC) to assess malnutrition in children aged 6–59 months.

Faine et al. conducted a systematic assessment of a random sample of 366 children residing in the Minawao refugee camp, Cameroon; this is the largest refugee camp in Cameroon, hosting over 60,000 Nigerian refugees displaced by the Boko Haram crisis [20]. In addition to collecting anthropometric data from children, sociodemographic data were collected from mothers using face-to-face questionnaires. The authors found an overall prevalence of 43.2% for undernutrition, 36.3% for underweight, 18.9% for wasting, 22.4% for stunting, and 2.7% for overweight. In the bivariate analyses, a large household size (>9 residents) was associated with wasting, and diseases such as diarrhea and respiratory tract infection were associated with underweight; however, none of the examined factors (mother’s age, marital status, level of education, household size, age of weaning, breastfeeding, or presence of diarrhea or respiratory tract infection) were significantly associated with stunting. The authors noted the prevalence of malnutrition was high compared to WHO standards, and suggested this was likely a consequence of inadequate food intake due to an increasing camp population leading to cuts in the food ration as well as the selling of food rations by some refugees to meet financial needs.

Using the same methodology, Ejigu et al. determined the prevalence and risk factors for malnutrition in a sample of 367 children in the Addi Harush refugee camp, Northern Ethiopia [18]. Additional data on immunizations, pregnancy and birth, infant feeding, and hygiene practices were collected in this study. Almost one in five (18.8%) children were affected by stunting, and 9.8% were affected by wasting. Children who were served food after hand washing were less likely to be stunted, while children who were bottle-fed were more likely to be stunted. Children who were exclusively breastfed for six months were less likely to be wasted, while children who had never been immunized were more likely to be either stunted or wasted. The authors noted that the prevalence of malnutrition in their study was lower than previous studies in other areas of Ethiopia and suggested that the use of consistent nutrition assessments in the camp may have enhanced nutritional knowledge and feeding practices.

An earlier study by Kelati et al., also conducted in Ethiopia, found a much higher prevalence of malnutrition [29]. In their systematic random sample of 593 children residing in the Mai-Aini Eritrean refugee camp, Northern Ethiopia, the prevalences of underweight and wasting were 33.4 and 24.6%, respectively [29]. These measures of acute malnutrition were more prevalent in male than female children. The authors also collected information on sociodemographic characteristics, household environmental health, birth order, child health problems, breastfeeding, hygiene, health and nutrition during pregnancy/lactation, and family planning. Using a multivariate analysis, the authors identified that children were more likely to be underweight if their mothers were underweight and less likely to be underweight if their mother consumed extra food during pregnancy or they were between the ages of 25 and 35 months. The odds of being wasted were higher among children who received pre-lacteal food (i.e., food other than breastmilk in the first three days after birth) and those aged 36–47 months. The authors highlighted the influence of maternal health and nutrition, as well as early feeding practices, on acute childhood malnutrition; however, the study did not examine chronic malnutrition in the form of stunting.

Renzaho and Renzaho conducted a similar study of acute malnutrition in the Katale refugee camp (now abandoned due to military attack) in Zaire (now DRC). In addition to anthropometric data, the authors collected data on immunization, mortality rates, and the food ration [35]. Among a sample of 431 children aged 6–59 months, recruited using two-stage cluster sampling, the overall prevalence of acute malnutrition was 3.5%. The prevalence of acute malnutrition in children 6 to 29 months old was seven times that of children 30 to 59 months old. The authors pointed out that mortality rates in the camp had significantly improved since the height of the Rwandan refugee crisis but malnutrition rates remained largely unchanged. In addition, their analysis of the general food ration highlighted its inadequacy, nutritional imbalance, and inequitable distribution.

Porignon et al. performed cross-sectional surveys in the DRC to compare the nutritional status of refugee children with that of local host populations, a year after significant population displacement caused by the outbreak of the Rwandan refugee crisis [34]. The authors used cluster sampling with probability proportional to the size to examine 2504 children living in five refugee camps near the town of Goma and 2617 children in neighbouring rural and urban health districts. The prevalences of stunting, underweight, and wasting in children within the refugee camps (50.9, 27.5, and 1.7%, respectively) were higher than those of children living in urban areas, but lower than those of children living in rural areas. The prevalence of edema was lower among the refugee children (0.4%) than in the urban and rural populations. The prevalence of stunting and underweight among the children in the refugee camps peaked in the 36- to 47-month age group, while wasting was highest in those aged 6 to 11 months. This pattern differed from that of the urban and rural children. The authors highlighted the possibility that vulnerable groups of the local host population may become ‘indirect’ victims of population displacement crises, and equitable nutritional relief must take this into account.

Two additional quantitative studies concerning malnutrition were included in this scoping review. A study by Grijalva-Eternod et al. examined the impact of a cash-based intervention on the incidence of acute malnutrition among children aged 6 to 59 months residing in camps for IDPs in Mogadishu, Somalia [24]. The intervention consisted of a monthly unconditional cash transfer of USD 84 (for five months), a once-off non-food-items kit, and free piped water. The authors made monthly assessments of acute malnutrition, including MUAC and the presence of edema, in 847 children in 10 camps receiving the intervention and 1379 children in 10 control camps. The sampling method was non-randomized and was based on vulnerability criteria. Using intention-to-treat analysis, the authors found the intervention did not influence the risk of acute malnutrition (incidence rate of 0.77 cases/100 child-months in the intervention group (95% CI 0.70; 1.21) and 0.92 in the control group (95% CI 0.53; 1.14)). A survey was carried out on 120 households in both the intervention and control groups to investigate factors contributing to malnutrition. This analysis included aspects such as household demographics, wealth, food security, morbidity, access to healthcare, water, sanitation, hygiene, and practices related to infant and young child feeding. The study revealed that the intervention led to enhancements in household wealth, food security, and the dietary diversity of children. Despite these positive outcomes, the authors recommended further research to comprehend the reasons behind the limited nutritional impact of the intervention and explore avenues for improvement.

Glew et al. conducted a quantitative observational study to investigate the impact of displacement on child growth and body composition [23]. Using an unspecified sampling method, the authors identified 30 Fulani children aged 4 to 13 years residing in an IDP camp in Toro, central Nigeria. The children were part of a larger nutritional survey conducted a month prior to an attack causing the Fulani population to flee to temporary camps. After 7 months of residing in the camp, the growth and body composition (i.e., fat and fat-free mass) of the children did not significantly deteriorate. The authors concluded that displacement in general may not necessarily have a negative impact on child growth. They highlighted possible protective factors such as adequate and reliable food supplies, not separating mothers and children, and being located in a secure region among people of similar culture/ethnicity.

Ajakaye et al. conducted a cross-sectional quantitative study utilizing anthropometric measurements from a cohort of 250 children within an IDP camp in Edo State, Nigeria [16]. The authors found that malnutrition was prevalent within the children evaluated (41.2%), followed by stunting (39.2%), underweight (11.2%), and wasting (0.04%). Malnutrition was most prevalent among children aged 0 to 5 years. Additionally, a higher percentage of males exhibited malnourishment, being underweight, and experiencing stunting (43.6%, 18.1%, and 42.6%, respectively) compared to females (39.7%, 7.1%, and 37.2%, respectively). These findings indicate public health concerns related to malnutrition for the under-5-years-old population, as well as gender disparities with an increased prevalence in males compared to females.

Fenn et al. analyzed data related to malnutrition in 12 refugee camps over a six-year period in Chad [21]. In the initial year of data collection (2010), nearly half (49.8%) of all children exhibited stunting, 10.6% showed signs of wasting, and 6.0% experienced both stunting and wasting. The prevalence of stunting among children aged 24 to 59 months (50.8%) surpassed that in the younger age group (6 to 23 months; 48.2%). However, the prevalence of wasting was higher in the younger age group (13.9% vs. 8.8%). Over time, with the introduction of fortified food sources, there was an average reduction of 13.0 percentage points (from 57.7% to 44.7%) in the prevalence of children classified as stunted or wasted from 2010 to 2017. This study demonstrated that the burden of wasting and stunting is a significant public health challenge within refugee camp settings. Even with an established food distribution, rations, and provision program, the study demonstrated that stunting and wasting impacts the growth and development of a significant proportion (44.7%) of children in the refugee camps analyzed.

Seal et al. conducted a prospective cohort study of 25 internal displacement camps (IDCs) within Somalia from March 2016 to March 2018 [37]. The study investigated the cause of death for children under 5 years of age. The third-leading cause of death within the study was severe malnutrition (8.8%). Younger children with a smaller Mid-Upper Arm Circumference (MUAC) were more prone to mortality compared to those who survived. This study demonstrates morbidity and mortality related to malnutrition continues to be a significant threat for children under 5 within IDCs.

Engidaw and Gebremariam also investigated Somali children residing in the Aw-Barre refugee camp in eastern Ethiopia [19]. Employing a cross-sectional approach, the investigation incorporated 415 adolescent girls, with an average age of 13.94 years, to assess the prevalences of stunting, thinness, and wasting. The study found a 1% prevalence for severe stunting, with a moderate stunting rate of 8.7%. Older adolescents (aged 15–19) were twice as likely to experience stunting compared to their younger counterparts. Additionally, premenarcheal adolescent girls had a 64% lower likelihood of being thin compared to post-menarche adolescents. The overall wasting prevalence was 15.2%, with 13% moderately wasted and 2.2% severely wasted. The majority of the participants had a good dietary diversity score (68.2%), while 27.2% had a medium score and 4.6% had a poor score. Overall, the authors concluded the prevalence of stunting was a low public health concern within this refugee camp; however, thinness and wasting were moderate public health concerns. Stunting was associated with age, and menarcheal status was linked with thinness.

Idowu et al. conducted a descriptive cross-sectional study comprising 317 mother–child (0–59 months) dyads in Abuja, Nigeria, within IDCs [25]. The data were obtained using pretest, semi-structured, and interviewer-administered questionnaires. The prevalences of underweight, stunting, and wasting were 42, 41, and 29.3%, respectively. The prevalence of underweight among children under five was 42%, with 9% being severely underweight; the prevalence was higher in male (46%) vs. female (37.8%) children. The prevalence of overall stunting was 41%, with 12.9% being severely stunted. The occurrence of stunting was also higher in males (44.7%) compared to females (37.2%). Lastly, the prevalence of wasting was 29.3%, of which 5.3% were severely wasted. However, wasting was more prevalent in female (29.5%) than male (29.2%) children. Malnutrition remains a major health problem among under-five children in IDCs, affecting approximately one in three children in this study. The key determinants of malnutrition in this population included age, birth order, gender, and deworming status.

Kiarie et al. conducted a cross-sectional study among 630 children aged 6–59 months from 348 households in Yambio County, South Sudan, from 26 October–6 November 2018 [30]. Within the sample of children, 2.5% were non-residents, 111 (86%) were internally displaced, and 15 (12%) were refugees. The children from households of non-residents were greater than four times more likely to be wasted than the nationalized residents of Yambio County.

Kalid et al. explored infant and young child nutritional status and their caregiver’s knowledge of pediatric nutritional needs in three selected IDCs within Somalia [27]. This study utilized a cross-sectional descriptive design, with questionnaires completed by households comprising a total of 2370 children. MUAC measurements were also carried out for all 2370 children. Severe malnutrition occurred in 12.1% of the participants and global acute malnutrition occurred in 19.9% of the participants. Children aged 6 to 24 months had a greater occurrence of malnutrition compared to the 25- to 59-month age group. Questionnaires from caregivers indicated that the risk factors for malnutrition were derived from gaps in caregiver knowledge about pediatric dietary needs, attitudes, and practices related to hygiene and feeding practices. The findings support the need for the development of educational programs for identified high-risk populations to address caregiver knowledge gaps related to nutrition.

One article focused on malnutrition using a mixed-methods study design. This study by Olwedo et al. was conducted across four IDP camps in Omoro County, Gulu District, northern Uganda [33]. Using multi-stage randomized cluster sampling, the authors collected anthropometric measurements and administered household surveys to determine the prevalence and risk factors for malnutrition in a sample of 672 children aged 3 to 59 months; they also conducted focus groups with parents and caregivers (*n* = 48) and interviews with camp leaders and health workers. The authors found that the prevalence of stunting was 52.1% and was higher among male children. The prevalence of wasting was 6.0% and was higher among children aged 3–24 months than those aged 25–59 months, but lower among children who had deworming treatment in the three months preceding the survey. Focus group discussions and interviews revealed contributing factors, such as inadequate quantity of food rations, the selling of food rations, difficulty accessing food rations for new IDPs, limited ability to cultivate land, and mothers needing to take up casual labour away from the home.

### 3.2. Malnutrition: Micronutrient Imbalances

Eight studies examined the micronutrient concerns among displaced children. Six of these investigated anemia.

Jemal and colleagues carried out a cross-sectional investigation involving 399 preschool children, randomly chosen from the age group of 6–59 months, within the Kebribeyah refugee camp, Somali region, Ethiopia [26]. The overall anemia prevalence stood at 52.4%. Moderate anemia was most common (36.6%); however, 1 in 10 (10.5%) children had severe anemia. Overall, 29.3% of children were stunted, 26.8% were underweight, and 10.3% were wasted. The authors also examined the risk factors for anemia, finding that increasing number of children under 5 years in the household, being underweight, and age 18–29 months were associated with increased odds of anemia in a multivariable analysis. Issues such as inadequate food rations, insufficient micronutrient supply in the ration, poor nutrition services, and poor sanitation were highlighted as factors contributing to high rates of anemia.

Ndemwa and colleagues carried out a prospective cohort study to assess the influence of micronutrient powder fortification on the prevalence of anemia [32]. Baseline, midline (6 months), and endline (13 months) blood samples of 410 children aged 6–59 months in the Kakuma refugee camp, Kenya, were collected along with anthropometric measurements and data from household questionnaires. No statistically significant change in the prevalence of anemia occurred over the course of the study (55.5, 52.3, and 59.8% at baseline, midline, and endline, respectively). The authors noted that compliance and loss to follow-up were significant issues in the study, as the proportion of adults collecting the micronutrient powder dropped to as low as 30% during the course of the study and almost 27% of children were lost to follow-up by the endline visit.

Ajakaye and colleagues conducted a study to examine the occurrence of anemia and malnutrition, along with the contributing risk factors, among children residing in an Internally Displaced Camp (IDC) in Edo State, Nigeria [16]. Using a cross-sectional survey, the study collected anthropometric measurements of the children within the study as well as blood samples for hematocrit readings. Overall, 250 children with a mean age of 7.1 years were assessed. Of these children, 135 (54%) had anemia, with 45 (18%) characterized as mild and 90 (36%) characterized as moderate. Children aged 6 to 10 years had a greater prevalence of anemia compared to those aged 0 to 5 years.

Adelman et al. conducted a randomized controlled study evaluating the impact of iron-fortified supplements given to children and adolescents within IDCs in Uganda [15]. The study considered 11 IDCs within Uganda and three study groups: control, school, and home. The school group received iron-fortified supplementation at school and the home group was provided supplements within the household. At baseline, the prevalence of anemia for girls aged 10–13 years was 40–46%, with moderate-to-severe anemia accounting for 23%. Within preschool children (6–59 months), anemia was evident in 69–72% of participants and moderate-to-severe levels were present in 38–51% of participants. The introduction of the iron-fortified supplements resulted in reduced moderate-to-severe anemia rates among adolescent females. Furthermore, a large reduction of moderate-to-severe anemia prevalence was evident in children aged 6–59 months living in IDP camps and attending schools. Overall, the study supports the use of iron-fortified supplementation programs, as these programs were seen as a protective factor against increases in anemia prevalence.

Another study sought to identify the prevalence of anemia in pre-settlement African asylum-seeking children in Israel [31]. Between 1 January and 30 June 2018, 386 eligible children were identified, of which 34% were anemic; this was four times the prevalence in Jewish toddlers and young children within the same age group. In a subgroup investigation on daily iron intake, 46.2% of asylum-seeking African children did not receive the recommended daily allowance for their age. The study indicated the need for routine assessment of anemia in asylum-seeking children to inform supportive strategies to increase iron dietary intake.

Kay et al. conducted a cross-sectional study examining the burden of anemia in displaced children aged 6–59 months [28]. The research analyzed information gathered between 2013 and 2016, encompassing 196 surveys involving children across 121 distinct refugee locations in 24 countries. West and Central Africa exhibited the highest prevalence of overall anemia in children at 58%, with the most elevated rates of moderate-to-severe anemia recorded at 32%. These data indicate the public health burden of anemia within internally displaced children in these regions.

Seal et al. sought to investigate the iodine status of long-term refugee children. Using a series of cross-sectional surveys, the study collected data on urinary iodine excretion, prevalence of visible goiters, and iodine concentrations in market-level and household-level salt samples among a sample of 895 children aged 10–19 years residing in six refugee camps located in Kenya, Uganda, Ethiopia, Algeria, and Zambia [36]. In five of these camps, excess iodine intake was evident as the median urinary iodine concentration exceeded the recommended maximum limit (300 µg L^−1^). The prevalence of visible goiters (indicating iodine-induced thyroid dysfunction) ranged from 0 to 7.1% and appeared to show a dose–response relationship with median urinary iodine concentrations. Of the 11 salt samples, 6 exceeded the WHO recommendations for iodine concentration. The authors emphasized the potential for adverse health effects arising from these excess levels of iodine and suggested an urgent need for further investigation and remedial action.

Finally, Di Marcantonio et al. conducted a study with participants from IDCs within Somalia, conducting face-to-face interviews from June 2014 to June 2015 to investigate the food diversity [17]. Food diversity was defined as children aged 6 to 23 months consuming four or more of the seven predefined food groups (FGs). The covariates investigated as food security proxies included the Household Dietary Diversity Score (HDDS), Food Consumption Score (FCS), and anthropometric indicators such as weight, height, and age. The findings from this study showed that 18% of participants were acutely malnourished and 29% were stunted. The global acute malnutrition was significantly higher in males (20.8%) compared to females (15.1%). Household food security status was found to be low overall. Most of the children only consumed two or three different FGs, with the most consumed FG being dairy. The consumption of fruits, vegetables, and eggs was found to be extremely low. The study also found that children staying in the IDC for a period of 1 to 3 years were less likely to achieve the minimum child dietary diversity (MDDC) relative to children staying in the IDC for a shorter period of 1 to 11 months.

### 3.3. Neonatal Health

The final study included in this scoping review used qualitative methods to explore newborn health in refugee camps. Gee et al. aimed to comprehend the needs of and potential enhancements for newborn health. They conducted focus group discussions involving 147 participants, including parents, grandmothers, birth attendants, midwives, and community health workers. Additionally, semi-structured interviews were conducted with 18 individuals, comprising health service managers, front-line providers, and public health officials, within the Doro and Yusuf Batil refugee camps in Maban County, South Sudan [22]. The factors associated with newborn health within the sociocultural and contextual context were recognized. These encompassed concerns regarding family planning, insufficient nutrition, limited livelihood opportunities, insecurity, material incentives for antenatal and intrapartum care, inadequate transportation, preference for home births, apprehensions about hospital deliveries, the role of traditional birth attendants, and harmful traditional newborn practices. The authors put forth recommendations to enhance newborn health in refugee camp environments. These suggestions included improved nutritional assistance, the implementation of community-based emergency transportation, permitting a birth companion, addressing detrimental practices during home-based newborn care, and enhancing the skills and knowledge of community health workers.

## 4. Discussion

The objective of this scoping review was to assess the scope, diversity, and characteristics of research efforts regarding the nutritional status and associated health outcomes of African refugee or displaced children before resettlement, with the additional goal of pinpointing any existing gaps in the literature. To our knowledge, this is the first synthesis of the literature on this topic in the form of a scoping review, providing valuable insight into the evidence available for policy, program, and research planning.

The articles included (*n* = 23) data on the health status of African refugee/IDP children from 1995 to 2018, spanning a period of 23 years. The large majority (*n* = 21) of the articles included in this scoping review used quantitative research methods. Only one qualitative study and one mixed-methods study were also included. These articles highlighted the wide variation in levels of malnutrition among African refugee/IDP children, with the prevalence of chronic malnutrition (stunting) ranging from 18.8 to 52.1% and from 0.04 to 29.3% for acute malnutrition (wasting). Chronic malnutrition was of ‘high’ or ‘very high’ severity according to recent WHO classifications [36] in 80% of studies, while acute malnutrition was of ‘high’ or ‘very high’ severity in 50% of studies. In addition, iodine levels were found to be excessive in 83% of the camps surveyed, while the prevalence of anemia, at 52.4 and 72%, was significantly higher than the 40% level considered to indicate a ‘severe public health problem’ [17]. Anemia in African children can stem from a variety of factors. These may include deficiencies in essential nutrients due to insufficient dietary intake or impaired nutrient absorption [32,38]. Additionally, infections, such as malaria, parasitic infections, tuberculosis, and HIV, can contribute to anemia, along with inflammation, chronic diseases, and hereditary red blood cell disorders [39]. Iron deficiency is the predominant nutritional factor leading to anemia, although deficiencies in folate, and vitamins B12 and A are also significant contributors [39]. However, no studies identified by this review examined other micronutrient deficiencies common in displaced populations, such as vitamins A, C, B, D, and niacin [40]. No studies examined the intestinal microbiota diversity or other aspects of the microbiome and the impact on nutritional status in the described populations.

While these quantitative studies provide critical data on the magnitude and scope of malnutrition, the gap in qualitative research impedes a deeper understanding of its nature and impacts on African refugee and displaced children and their communities. The focus of the qualitative investigation in the articles included in this review were neonatal health and food accessibility. Future qualitative research could build on this knowledge by exploring the tangible and intangible factors that influence the nutrition of children up to five years of age in refugee/IDP settings, for example, community and family structure, food acceptability and accessibility, gender roles, and cultural expectations.

Most (*n* = 17) of the articles included in this scoping review specifically focused on children under 5 years of age. Only five articles included older children (4–19 years), and one article exclusively focused on newborn health. About 40% of refugees and asylum seekers in Africa are between 5 and 17 years old [1], and although malnutrition-related mortality among all children in Sub-Saharan Africa is much lower in those aged 5 to 14 years compared to those aged under 5 years [41], it is unclear whether this is also the case for children affected by displacement. This emphasis on children aged 6–59 months could be due to the critical window for child development found in this category, spanning the first 1000 days of life from conception to 2 years [4]. Also, this focus is particularly relevant during the period of complementary feeding and early childhood, which is crucial for brain development [4,5]. Nonetheless, some studies reported results on other age groups. This approach ensures a comprehensive exploration of child development within the specified age range while acknowledging variations in research focus. Future research could include broader age ranges or specifically target older children to address this gap in the literature.

This scoping review aimed to include studies of refugee and IDP children throughout Africa. Although many countries in the Sub-Saharan region were represented, the articles included predominantly focused on countries in East Africa, including Ethiopia, Kenya, Somalia, South Sudan, and Uganda. A smaller number of studies were conducted in Central, Southern, and West African countries, including Algeria, Zambia, Cameroon, Nigeria, and the DRC. Countries such as Burkina Faso, Mali, Mauritania, Niger, Central African Republic, Burundi, and Eritrea host significant numbers of refugees and IDPs [2] but are not represented in the literature. Without further research, the nutritional challenges and needs of displaced children in these areas remains uncertain.

Although not included in the review but of relevance is Cumber et al.’s commentary on the nutritional status of refugee and internally displaced children in Cameroon resulting from the Boko Haram conflict [12]. The authors highlighted the large numbers of malnourished children in Cameroon refugee camps, and a growing influx of displaced people caused by ongoing conflict and worsened by natural disasters. They further argue that the humanitarian response in the area has been limited and called for greater financial and material support for medical treatment of severe malnutrition in refugee camps, increased food access, education for caregivers, aid for mothers, and family planning education.

Only four studies included in this scoping review examined the effect of an intervention designed to address malnutrition among refugee/IDP children. The first examined a cash-based intervention [24], while the second evaluated micronutrient powder fortification [32]. The third examined lipid-based nutritional supplementation [21], and the fourth evaluated micronutrient-fortified meals provided in schools and homes [15]. These interventions are widely used and have been shown to have positive effects in various populations around the world in systematic reviews [38,39]. However, the two studies [24,32] found little evidence of a positive effect for children under 5 years of age living in refugee or IDP camps in Somalia and Kenya. There is a clear need for further research on a broader range of interventions to address malnutrition and how such interventions may be successfully applied in the specific context of refugee and IDP children. Future research could incorporate both nutrition-specific programs as well as nutrition-sensitive interventions in education, early childhood development, social safety nets, and agriculture, which have the potential to complement and enhance traditional programs [40].

Our scoping review should be considered with the following limitations in mind. We focused on English-language literature available electronically in the described databases, and therefore may have excluded relevant articles not available in this format. Searching a wider range of databases and including grey literature could have identified additional relevant articles. The studies included spanned a period of twenty-three years, and within that time frame, various historical or political events or societal changes may have occurred. These contextual factors may introduce confounding variables impacting the interpretation of the results. Nonetheless, our review incorporated articles with a range of study designs and methodologies and provides valuable insight into the state of the literature, spanning twenty-three years, on the nutritional needs of African refugee/displaced children prior to resettlement.

Finally, this scoping review analyzed articles published from January 2000 to October 2021, encompassing the COVID-19 pandemic, which emerged in 2019. The pandemic posed new obstacles to child nutrition worldwide, including those in Africa. Lockdowns, economic disruptions, and healthcare constraints may have hindered the ability of African children who are refugees or internally displaced to access food and healthcare services. The pandemic led to significant disruptions in global food supply chains, resulting in food shortages and increased prices. The loss of income due to lockdowns may have compounded the problem of food insecurity and malnutrition of African children who are refugees or internally displaced. Additionally, the increased strain on healthcare systems may have caused a reduction in routine healthcare services, such as nutritional check-ups, that were available. School closures may have also affected the delivery and availability of nutritional programs, impacting the nutritional status of African refugees and displaced children.

In conclusion, this scoping review has identified the extent, range, and nature of research activity on the nutritional status and associated health outcomes of African refugee/displaced children prior to resettlement. A wide variation in the prevalence of acute and chronic malnutrition was identified and, in many settings, acute, chronic, and micronutrient malnutrition were at levels of great concern. However, many countries hosting large, displaced populations are not represented in the literature, and research among older children is also lacking. UNICEF has reported that 43% of African countries and territories lacking age-disaggregated data on migrants [41]. There is a dearth of qualitative research on this topic, and further research into effective nutrition interventions for this population group is urgently needed.

## 5. Practical Implications and Policy Recommendations

The findings of this extensive scoping review carry substantial implications for the practical dimensions of nutrition and healthcare provision for African refugee and internally displaced children, necessitating explicit considerations for potential policy changes. The documented prevalence of malnutrition, spanning stunting, wasting, and underweight conditions underscore the critical need for targeted nutritional interventions tailored to the unique challenges faced by these populations. It is imperative that such initiatives go beyond addressing mere quantity and also prioritize the quality of food, and recognizing and catering to the specific nutritional requirements of children in these challenging environments.

Additionally, this study emphasizes the urgency of targeted micronutrient supplementation, particularly in addressing prevalent issues such as anemia and other micronutrient imbalances. The identified gaps in caregiver knowledge regarding infant and young child dietary needs underscore the vital role of nutrition education and awareness programs, specifically targeted at caregivers within these communities.

To effectively combat the morbidity and mortality associated with malnutrition, there is a pressing need for improved healthcare infrastructure, enhanced accessibility, and the integration of mental health support. Long-term monitoring and evaluation of nutritional programs, advocacy for sufficient food rations, collaborative efforts among stakeholders, and policy adjustments are crucial components for ensuring sustained and effective interventions to enhance the health and well-being of African refugee and internally displaced children.

Policymakers should consider endorsing policies that enhance healthcare infrastructure and accessibility, integrating mental health support into existing frameworks. Advocacy efforts should focus on securing adequate food rations for these vulnerable populations. Furthermore, collaborative initiatives among stakeholders are essential to fostering coordination and shared responsibilities.

Policy changes should be directed towards establishing a comprehensive framework for long-term monitoring and evaluation of nutritional programs, ensuring their effectiveness and adaptability. These efforts can be strengthened through a commitment to continuous collaboration among various stakeholders, including governmental bodies, non-governmental organizations, and international agencies.

## 6. Limitations

Several limitations should be acknowledged in interpreting the findings of this scoping review. Firstly, the search strategy may have introduced selection bias, as only articles published in English were included, potentially excluding relevant studies published in other languages. Additionally, the search was conducted up until the knowledge cutoff date of January 2022. Consequently, there is a possibility that more recent studies or emerging trends beyond this date may not have been captured in the analysis, which may have potential implications on the comprehensiveness and currency of the findings. The exclusion of certain study types, such as conference abstracts and proceedings, may have led to the omission of valuable data. Furthermore, the focus on children aged 0 to 18 years might overlook critical information related to specific age groups within this range. The exclusion of resettled children and local populations may limit the generalizability of the findings to these groups. Also, it is crucial to acknowledge the observed gap in geographic representation, particularly the underrepresentation of countries hosting large, displaced populations due to lack of studies from these countries. We recognize that this limitation may impact the generalizability of our findings. The methodological variations among the included studies, such as different sampling methods and measurement tools, could introduce heterogeneity in the interpretation of the results. The predominantly quantitative nature of the included studies may underscore potential gaps in qualitative insights into the experiences of these populations. Despite these limitations, this scoping review offers a comprehensive overview of the existing literature on the health and nutrition of African refugee and internally displaced children, providing valuable insights for future research and practice.

## Figures and Tables

**Table 1:** Summary of articles included in this scoping review.

Article	Research Question/Objective/Aim	Methodology	Methods/Data Source/Age	Setting	Period of Data Collection	Sampling/Sample Size	Summary of Findings
Adelman 2019 [15]	To test whether food fortified with multiple micronutrients provided in FFE programs reduced anemia prevalence of primary-school-age adolescent girls, adult women, and preschool children	Quantitative cluster randomized controlled trial	Children aged 6–59 mo Adolescents aged 10–13 years old	31 IDP camps in Uganda randomly assigned to 3 groups: SFP, THR, and control	2005–2007	A survey of households (*n* = 627) 233 adolescent girls aged 10–13 222 children aged 6–59 mo	Among adolescent girls (aged 10–13 years) at the beginning of the study, the prevalence of any anemia ranged from 40% to 46%, while moderate-to-severe anemia affected 21% to 23% of girls. The prevalence of anemia was notably high in preschool children at the study’s commencement, with 69% to 72% having any anemia, and 38% to 51% experiencing moderate-to-severe anemia. In adolescent girls aged 10–13 years who were exposed to Food for Education (FFE) programs, there was a substantial 24 percentage points reduction in the prevalence of any anemia compared to the control group (*p* < 0.05). Additionally, in FFE schools, there was a significant (*p* < 0.05) decrease of 25.7 percentage points (95% CI: −0.43, −0.08) in the prevalence of any anemia, as measured by hemoglobin levels.
Ajakaye 2019 [16]	To determine the prevalence of malaria, anemia, malnutrition, and infection as well as the risk factors of these public health issues among children in IDP camp in Benin, Nigeria	Quantitative cross-sectional	3 months to 10 years	Benin, Nigeria, IDC camp	2018	250	Out of the total, 135 children (54%) were identified as having anemia. Among them, 45 (18%) had mild anemia and 90 (36%) had moderate anemia, with no instances of severe anemia reported in the study. The variations in anemia prevalence were statistically significant across different age groups, notably higher in the 6–10 years group compared to the 0–5 years group (*p* < 0.05). In terms of malnutrition, underweight, and stunting, a higher percentage of males (43.6%, 18.1%, and 42.6%, respectively) were observed compared to females (39.7%, 7.1%, and 37.2%, respectively), although the differences were not statistically significant in malnutrition and stunting. However, the prevalence of underweight exhibited a significant difference between genders (*p* = 0.025), with males having a higher prevalence.
Di Marcantonio 2020 [17]	To assess the dietary diversity and identify the factors associated with it among children (6–23 months) in Somalian IDP camps	Quantitative cross-sectional survey	Children aged 6 to 23 months	11 IDP camps in Somalia	June 2014 and June 2015	3188 children	Around 15% of children in IDP camps reached the minimum dietary diversity. Overall, our results confirm that not only are food security proxies the factors most associated with MDDC, but HDDS performs better than FCS. In addition, the results identified women as key decision-makers in the household, duration of household permanence in the settlement, women’s physiological status, frequency of milk feeding to child, type of toilet, and measles vaccination as positively associated with MDDC.
Ejigu 2017 [18]	To determine the prevalence and associated factors of malnutrition among children aged 6–59 months at Addi Harush refugees camp in 2015	Quantitative, cross-sectional	Anthropometric measurements, structured interview questionnaire Children, caregiver 6–59 months	Addi Harush refugee camp, Tigray region, Northern Ethiopia	March–June 2015	Systematic random sampling (households) and lottery method (from multiple household candidates) 367	There was a 18.8% prevalence for stunting and 9.8% for wasting. Children who were exclusively breast fed for the duration of six months were 0.04 times less likely to be wasted. Children who were bottle fed were 6.067 times more likely to be affected with acute malnutrition (wasting). Children who had been vaccinated with BCG were 0.037 times less likely to be wasted. Generally immunized children were 0.013 times less likely to be wasted and 0.054 times less likely to be stunted.
Engidaw 2019 [19]	To explore stunting and thinness and their associated factors among adolescent refugee girls	Quantitative cross-sectional study design	10–19 years	Aw-Barre refugee Camp, Ethiopia	February to March 2015	A total of 415 adolescent girls were included in the study	The study encompassed a total of 415 adolescent girls, yielding a response rate of 98.1%. The overall prevalence of stunting and thinness stood at 9.7% (95% CI: 7.0, 12.3) and 15.2% (95% CI: 11.8, 18.9), respectively. Older adolescent girls were 2 times more likely to experience stunting (AOR: 2.10, 95% CI: 1.12, 3.93) compared to their younger counterparts. Premenarcheal adolescent girls were 64% less likely to be thin (AOR: 0.36, 95% CI: 0.12, 0.75) in comparison to post-menarche girls. The prevalence of stunting and thinness among adolescent refugee girls was deemed a low and moderate public health concern, respectively. Stunting was significantly associated with age, while thinness was linked to the menarcheal status of the adolescent girls.
Faine 2018 [20]	To determine the prevalence and risk factors associated with malnutrition among children aged 6–59 months old in Minawao refugee camp, Cameroon	Quantitative, cross-sectional	Anthropometric measurements, structured interview questionnaire Children, mothers 6–59 months	Minawao refugee camp, Cameroon	March–April 2017	Systematic random sampling (households) 366	The prevalence of malnutrition among children aged 6–59 months in the Minawao refugee camp was notably high and reached a critical level. Out of the 366 children surveyed, the overall prevalence of undernutrition was 43.2%, with 158 children exhibiting at least one of the three indices (weight for height, weight for age, and height for age) below a Z-score of −2. Additionally, 2.7% of children had Z-scores exceeding 2 for either weight for age or height for age. The prevalence rates for underweight, wasting, and stunting were 36.3%, 18.9%, and 22.4%, respectively. Notably, a larger household size was linked to wasting (*p* = 0.038), while diseases showed an association with underweight (*p* = 0.005).
Fenn 2021 [21]	To use the United Nations High Commissioner for Refugees Standardised Expanded Nutrition Survey data to evaluate the effect of a change in food rations on child growth in refugee camps in eastern Chad	Quantitative cross sectional	6–59 months	12 refugee camps in eastern Chad	Between 2010 and 2017	39,231 records analyzed Children with missing data on both height and weight (*n* = 615) were dropped from the analysis. *n* = 14 children with missing weight data and *n* = 31 with missing height data. *n* = 67 datapoints were recorded as missing with non-plausible HAZ and 55 for non-plausible WHZ	Before 2014, the camps had access to a complete General Food Distribution (GFD), providing 2100 kcal per person per day, which was estimated to fulfill the energy requirements for children aged 6–23 months (900 kcal/day). However, in 2014, the GDF ration was reduced by 40–60% compared to the full ration. Starting in 2014, the UNHCR, in collaboration with the World Food Programme (WFP) and other partners, implemented a nutrition treatment and prevention package for pregnant and lactating women and children under 5 years of age in all eastern Chad camps, offering approximately 470 and 650 kcal/day. This represented a 24–48% reduction in energy from the pre-2014 ration. Overall, there was a significant decrease in the prevalence of stunting and wasting over time. The odds of experiencing both stunting and wasting were 1.38 times higher than having either condition separately (*p* < 0.001, 95% CI = 1.29 to 1.47). Trends in mean Height-for-Age Z-score (HAZ) and Weight-for-Height Z-score (WHZ) before and after the ration change in 2014 suggest that growth either slowed down or worsened. Following the ration change, children aged 24–59 months experienced a significant decrease in mean HAZ of 0.04 per year (*p* = 0.02, 95% CI = −0.07 to –0.01), and for the younger age group, there was a significant decrease in mean WHZ of 0.06 per year (*p* = 0.03, 95% CI = −0.12 to –0.01).
Gee 2018 [22]	To better understand key needs and opportunities to improve newborn health in refugee camp settings	Qualitative, cross-sectional	Semi-structured interviews and focus groups Interviews: ‘key informants’ [health program managers (*n* = 5), front-line health workers (*n* = 8), UN public health officials (*n* = 5)] Focus groups: mothers (*n* = 44), fathers (*n* = 22), grandmothers (*n* = 22), community health workers (*n* = 27), midwives (*n* = 8) Newborns (interviews with relevant adults)	Doro and Yusuf Batil refugee camps in Maban County, South Sudan	February 2016	Non-probabilistic; purposive and convenience (interviews) Word-of-mouth and purposive (focus groups)—not snowballing as it was NGO partners using word of mouth to recruit 18 (interviews); 147 (focus groups)	A number of sociocultural and contextual factors related to newborn health were identified, including family planning concerns, poor nutrition, lack of livelihood opportunities, insecurity, material incentives for antenatal and intrapartum care, poor transportation options, familiarity with home birth, fears of birthing in hospital, role of traditional birth attendants, and harmful traditional newborn practices.
Glew 2003 [23]	To analyze the changes in growth and body composition occurring among Fulani children following displacement into a temporary camp due to ethnic/religious violence	Quantitative, observational	Anthropometric measurements and body composition analysis of pre- and post-displacement children 4–13 years	IDP camp in Toro, east of Jos, Nigeria	August 2001 and April 2002	Not specified 30	Neither the growth nor body composition of the Fulani children deteriorated significantly following the crisis. In terms of mean values and relative to growth curves established during the tranquil period immediately preceding the crisis, all but one of the girls grew taller and gained more weight than predicted; two-thirds of the weight gained by the girls was due to fat. With regard to the male subjects, while they grew taller, they gained, on average, 30 percent less in height than predicted. However, the boys did gain 50 percent more weight than predicted. Unexpectedly, fat accounted for one-half or more of the weight gain in both the boys and girls. In general, the boys did less well than the girls in the months following the crisis. The phase angle of all subjects did not decline significantly during the interval pre- and post-crisis assessments.
Grijalva-Eternod 2018 [24]	To understand whether a cash-based intervention (CBI) would reduce acute malnutrition and its risk factors	Quantitative, intervention trial	Anthropometric measurements and household questionnaires Intervention consisted of unconditional cash transfer, once-off non-food items kit, and free piped water Children, mothers, primary carers 6–59 months	20 IDP camps in peri-urban Mogadishu, Somalia (10 intervention/10 control)	March–November 2016	Non-randomized (purposive, based on vulnerability criteria) cluster sampling 240 households; 2337 children	The Cash-Based Intervention (CBI) seemed to enhance the wealth and food security of beneficiaries but did not demonstrate a noticeable reduction in the risk of acute malnutrition among children in the Internally Displaced Person (IDP) camp. Within the household cohort, the CBI was associated with an increase in the Child Dietary Diversity Score by 0.53 (95% CI 0.01; 1.05). In the child cohort, the incidence rate of acute malnutrition (cases/100 child months) was 0.77 (95% CI 0.70; 1.21) in the intervention arm and 0.92 (95% CI 0.53; 1.14) in the control arm. The CBI did not appear to diminish the risk of acute malnutrition, with an unadjusted hazard ratio of 0.83 (95% CI 0.48; 1.42) and an adjusted hazard ratio for age and sex of 0.94 (95% CI 0.51; 1.74). The CBI seemed to result in an increase in monthly household expenditure of USD 29.60 (95% CI 3.51; 55.68), an elevation of the household Food Consumption Score of 14.8 (95% CI 4.83; 24.8), and a reduction in the Reduced Coping Strategies Index of 11.6 (95% CI 17.5; 5.96).
Idowu 2020 [25]	To identify the determinants of anthropometric indices among under-five children in internally displaced persons’ camps in Abuja, Nigeria	Quantitative cross-sectional study	Infants/children aged 0–59 months	Three sampled IDP camps in Abuja, Nigeria	Not specified	317 mother–child (0–59 months) pairs	The median age was 24 months, with 50.8% being male, and 42.3% were delivered at a health facility. Exclusive breastfeeding was reported for only 45.4%, while 28.8% were introduced to complementary foods too early. Deworming in the preceding six months was performed for 45.4% of the children, and 43.9% had complete and up-to-date immunization. The prevalences of underweight, stunting, and wasting were 42%, 41%, and 29.3%, respectively. Poor anthropometric indices were more common among male children, except for wasting. Having good anthropometric indices was 2.5 times more likely among children under 12 months than those aged 37 months or older (CI: 1.08–5.8), 2.4 times more likely among first-born children than fifth-born children (CI: 0.19–0.93), and 1.7 times more likely among female children than male children (CI: 1.08–2.82).
Jemal 2017 [26]	To assess the magnitude and contributing factors of anemia among refugee preschool children of the Kebribeyah refugee camp	Quantitative, cross-sectional	Anthropometric measurements, finger-prick blood test, questionnaire Children 6–59 months	Kebribeyah refugee camp, Somali region, Ethiopia	March 2010	Simple random sampling 399	The prevalence of anemia was 52.4%. Most of the anemic children, 36.6%, were classified as having moderate (Hb 7–9.9 gm/dL), followed by severe, 10.5%, (Hb < 7 gm/dL) while the remaining 5.3% had mild anemia (Hb 10–10.9 gm/dL). The prevalences of stunting, underweight, and wasting among those children who had a hemoglobin measurement were 29.3%, 26.8%, and 10.3%, respectively (Figure 1). Anemia was prevalent in all age groups but was highest among children aged 18–29 months. The age of the child, paternal educational level, number of children younger than five years of age in the household, sharing/selling part of ration, inadequacy of ration stock, presence of diarrhea, personal hygiene of the child, stunting, and underweight were significantly associated with anemia.
Kalid 2019 [27]	(1) To explore the nutritional situation of all children aged 6–59 months enrolled in a nutrition program provided by Save the Children in IDP camps; (2) to identify gaps in the caregivers’ hygiene and feeding practices	* Not stated: The described research design is a cross-sectional study	Caregiver-reported questionnaire responses, naturalistic observations of hygiene practices, and anthropometric measurements Children 6–59 months	Three IDP camps in three different districts in Somalia. The three selected IDP camps are located in the districts of Baidoa, Dharkenley, and Dayniile, Somalia.	August 2017	1655 caregivers for 2370 children aged 6 to 59 months	There was a high prevalence of severe malnutrition (12.1%) and global acute malnutrition (19.9%) observed in children enrolled in the nutrition program, with a higher frequency in the 6–24-month age group compared to the 25–59-month age group (*p* < 0.01). Additionally, household practices were generally below hygienic standards. Furthermore, caregivers demonstrated poor knowledge regarding the benefits of breastfeeding and appropriate complementary foods. Child malnutrition could potentially be linked to deficiencies in caregivers’ knowledge, attitudes, and practices related to hygiene and infant feeding.
Kay 2019 [28]	To assess global patterns in anemia among children aged 6–59 months and non-pregnant women of reproductive age living in refugee settings between 2013 and 2016	Quantitative cross-sectional and two stage cluster survey	Children 6–59 months and non-pregnant women of reproductive age	121 unique refugee settings in 24 countries	Between 2013 and 2016	196 surveys among children; 184 surveys among women	The median prevalence of overall anemia was around 55% higher, and the mean hemoglobin levels were 6 g/L lower among children aged 6–23 months when compared to children aged 24–59 months. In both women and children, the West and Central Africa regions exhibited the highest median prevalence of anemia. There is a pressing need for sustained, multisectoral efforts to decrease anemia, with a particular emphasis on children under 2 years of age and in refugee settings within the West and Central Africa regions.
Kelati 2014 [29]	To assess the prevalence of acute malnutrition and its associated factors, among children aged 6–59 months in Mai-Aini Eritrean refugee camp, Northern Ethiopia, in 2014	Quantitative, cross-sectional	Anthropometric measurements, structured interview questionnaire Children, mother 6–59 months	Mai-Aini Eritrean Refugee camps, Tigray region, Northern Ethiopia	1–30 January 2014	Systematic random sampling (households) and lottery method (from multiple household candidates) 593 mother–child pairs	Approximately 33.4% and 24.6% of children were underweight and wasted, respectively. The prevalence of acute malnutrition was higher in males compared to females. Underweight was associated with child age, consuming extra food during pregnancy, and a maternal BMI less than 18.5 kg/m^2^. Child age and receiving pre-lactate food were independently associated with wasting.
Kiarie 2021 [30]	To determine the prevalence and identify the sociodemographic, maternal, and child predictors of wasting, underweight, and stunting among children aged 6 to 59 months in Yambio County, South Sudan	Quantitative cross-sectional study. The survey applied a two-stage cluster sampling design	6–59 months	Yambio County, South Sudan	26 October to 6 November 2018	630 children aged 6–59 months from 348 households	The wasting prevalence was very low, underweight prevalence was low, and stunting prevalence was high. The impact of the 2016 conflicts that lead to large displacements may not have greatly affected under-five undernutrition. Interventions targeted at improving food diversity, increasing nutrition knowledge, and enhancing resilience in male children might reduce undernutrition.
Koren 2019 [31]	(1) To quantify the prevalence of anemia among African asylum-seeking children treated in the Terem Clinic for refugees in Tel Aviv; (2) to compare it to the rates among Jewish Israeli children; (3) and to correlate it with their nutritional iron intake	Quantitative retrospective cross-sectional design	All children aged 9 months to 12 years	Pre-resettled African Asylum seekers in Israel	1 January and 30 June 2018	386	The average age of the children (standard deviation) was 2.96 years (SD 2.77), and the mean hemoglobin level was 10.88 g/dL (1.47). Out of 386 eligible children, 131 (34%) were found to be anemic, which is four times more prevalent than the reported anemia rate among 263 Jewish toddlers and young children of the same age group (11%) with an odds ratio of 4.15 (95% CI 2.67–6.43). In a subgroup of 26 children investigated for their daily iron intake, 46.2% did not receive the recommended daily allowance for their age, and 9 of them had received iron supplements.
Ndemwa 2011 [32]	To evaluate the effect of the availability of home fortification with a micronutrient powder containing 2.5 mg sodium iron ethylenediaminetetraacetate (NaFeEDTA) on iron status and hemoglobin in women and children in the Kakuma Refugee Camp in northwest Kenya	Quantitative, prospective cohort	Blood samples at baseline, midline (6 months), and endline (13 months); anthropometric measurements, questionnaires Children, women 6–59 months	Kakuma refugee camp, northwest Kenya	January 2009–January 2010	Cluster sampling (probability proportional to size) 410 children; 458 women	In children, the average hemoglobin concentration was 105.7 ± 0.6, 109.0 ± 1.5, and 105.5 ± 0.3 g/L at the baseline, midline, and endline visits, respectively (*p* = 0.95). The prevalence of anemia (hemoglobin < 110 g/L) was 55.5%, 52.3%, and 59.8% (*p* = 0.26). Additionally, the mean soluble transferrin receptor concentration was 36.1 ± 0.7, 29.5 ± 1.9, and 28.4 ± 3.2 nmol/L (*p* = 0.02) at the corresponding visits. These values were derived from models adjusted for age using least squares means regression.
Olwedo 2008 [33]	To estimate the prevalence of and describe the risk factors for protein energy malnutrition among children under five years old living in internally displaced persons camps in Omoro County, Gulu District	Mixed methods, cross-sectional	Anthropometric measurements, interviews, focus group discussions Children, caregivers, key informants 3–59 months	4 IDP camps in Omoro county, Gulu District, northern Uganda	13–23 September 2006	Multi-stage randomized cluster sampling; households selected using modified WHO cluster sampling (probability proportional to size) from randomly selected IDP camps 672 children 48 mothers, fathers, and caretakers in 6 focus groups	The prevalence of stunting was found to be 52.4% and 6.0% for acute malnutrition. Male children were at risk of being stunted (adjusted OR 1.57 95% CI 1.15–2.13; *p* value = 0.004). Children in the 3–24-month age group were at risk of acute malnutrition (adjusted OR 2.78 95% CI 1.26–6.15; *p* value = 0.012) while de-worming was protective (adjusted OR 0.44 95% CI 0.22–0.88; *p* value = 0.018).
Porignon 2000 [34]	To compare children’s nutritional status in refugee populations with that of local host populations, one year after outbreak refugee crisis in the North Kivu region of Democratic Republic of Congo	Quantitative, cross-sectional	Anthropometric measurements children 6–59 months	Five refugee camps in the vicinity of the town of Goma, two neighbouring rural health districts (Kirotshe and Masisi), and the urban health district of Goma, DRC	June to August 1995	Cluster sampling (probability proportional to size) 5121	Children in all locations demonstrated a typical pattern of growth deficit relative to the international reference. The prevalence of acute malnutrition (wt/ht < −2 Z score) was higher among children in the rural non-refugee populations (3.8 and 5.8%) than among those in the urban non-refugee populations (1.4%) or in the refugee population living in temporary settlements (1.7%). The presence of edema was scarcely noticed in the camps (0.4%) while it was a common observation at least in the most remote rural areas (10.1%). Compared with the baseline data collected in 1989, there was evidence that the nutritional status was worsening in rural non-refugee populations.
Renzaho 2003 [35]	To evaluate the public health and nutritional situation of refugee children in Katale camp, Eastern Zaire, after two years of nutritional and health intervention	quantitative, cross-sectional	Anthropometric measurements, immunisation data collected children 6–59 months	Katale refugee camp, Zaire	May–June 1996	Two-stage cluster sampling; probability proportional to population size 431	Malnutrition was most prevalent in children aged 6–29 months old (6.2%) compared to the overall malnutrition prevalence (3.5%). The general food ration provided 6240 kJ on average per person per day, meeting only 57% to 84% of the minimum adult energy requirements. Measles immunization coverage in children aged 9–59 months was 88.6%. The crude mortality rate was 0.3 per 10,000 per day. The factors that contributed to malnutrition were inadequate food ration due to food shortages, lack of ability to supplement the diet due to economic restrictions imposed in the camp, and inequities in the food distribution process due to food being siphoned off by camp leaders for military purposes.
Seal 2006 [36]	To assess the iodine status of long-term refugees dependent on international food aid and humanitarian assistance	Quantitative, cross-sectional	Visual goiter assessment, analysis of urinary iodine in urine samples, analysis of iodine concentrations in market-level and household-level salt samples Children 10–19 years	Long-term refugee camps in Kenya, Uganda, Ethiopia, Algeria, and Zambia	March 2001–July 2003	Two-stage randomized cluster sampling; systematic sampling (Nangweshi camp, Zambia) 895	The median urinary iodine concentration (UIC) varied from 254 to 1200 µg/L, and in five of the camps, it surpassed the recommended maximum limit of 300 µg/L, indicating excessive iodine intake. The assessment of visible goiters was conducted in four surveys, with the prevalence ranging from 0.0 to 7.1%. Notably, the camp with the highest UIC also exhibited the highest prevalence of visible goiters. The iodine concentrations in 11 salt samples from three camps were measured through titration, and six of these exceeded the production-level concentration of 20 to 40 ppm recommended by the World Health Organization (WHO), although all were below 100 ppm.
Seal 2021 [37]	To provide evidence on the causes of death among children younger than 5 years in camps for internally displaced people in southern Somalia during periods of protracted displacement and emergency influx amid the 2017 drought and health emergency	Quantitative prospective cohort study	Infants/children aged 6 to 59 months	25 camps in the Afgooye corridor, on the outskirts of Mogadishu, Somalia	March 2016–2018	3898 children	Between March 2016 and March 2018, a total of 3898 children were monitored. Throughout 34,746 person-months of observation, 153 deaths were documented. The mortality rate for children under 5 years surpassed emergency thresholds (>2 deaths per 10,000 children per day), peaking at seven deaths per 10,000 children per day during the emergency influx. Verbal autopsy data were obtained for 80% of the recorded deaths, revealing that the Cause-Specific Mortality Fraction (CSMF) for the three primary causes of death were diarrheal diseases (25.9%), measles (17.8%), and severe malnutrition (8.8%). The elevated mortality rate from infectious diseases and malnutrition among children under 5 years underscores the necessity for reinforcing various public health interventions.

## Data Availability

The data presented in this study are available in article.
